# Cytokine profiles and laboratory parameters as indicators to distinguish children with PFAPA and bacterial infection

**DOI:** 10.3389/fimmu.2026.1683221

**Published:** 2026-02-12

**Authors:** Xiaona Zhu, Zhi Yang, Yanyan Huang, Ying Luo, Jun Yang, Tingyan He

**Affiliations:** Department of Rheumatology and Immunology, Shenzhen Children’s Hospital Affiliated Shantou University Medical College, Shantou University, Shenzhen, China

**Keywords:** bacterial infection, cytokines, IFN-γ, PFAPA syndrome, tonsillitis

## Abstract

**Objective:**

Periodic fever, aphthous stomatitis, pharyngitis, and cervical adenitis syndrome (PFAPA) is characterized by recurrent febrile episodes associated with one or more of the symptoms described by the acronym, and easily misdiagnosed as other infectious diseases, especially tonsillitis. We aimed to describe the clinical, laboratory parameters and cytokine profiles in patients with PFAPA and to explore indicators to distinguish children with PFAPA from bacterial infection.

**Methods:**

Patients with PFAPA and bacterial infection, who had cytokine panels performed by Flowcytomix technique during the febrile episodes (prior to any treatments), were retrospectively enrolled from January 2020 to June 2024 in Shenzhen Children’s Hospital. Clinical data were collected from inpatient medical records. Serum levels of cytokines and other laboratory parameters were compared between patients with PFAPA and those with identified bacterial infection. Multivariate regression analysis and a receiver operating characteristic (ROC) curve analysis were performed to construct a diagnostic model to assess the potential role of serum cytokines and laboratory parameters in the diagnosis of PFAPA.

**Results:**

67 patients with PFAPA and 160 patients with identified bacterial infection were included in this study. The level of serum IFN-γ, and the IFN-γ/IL-6 ratio in PFAPA patients were significantly higher than those in identified bacterial infection (*p* < 0.0001). The cutoff value of serum IFN-γ/IL-6 ratio for differentiating PFAPA from bacterial infection was > 0.43, and the area under the receiver operating characteristic curve (AUC) was 0.79, with a sensitivity of 70.15% and a specificity of 71.88%. A diagnosis model combined IL-10, platelet and IFN-γ/IL-6 ratio was built, and the AUC was 0.95, with the sensitivity and specificity as 90.3% and 89.6%, respectively.

**Conclusions:**

The IFN-γ/IL-6 ratio during febrile episodes may be useful in the early diagnosis of PFAPA by differentiating this disease from bacterial infection. The combined model based on IFN-γ/IL-6, IL-10, and PLT optimizes the diagnosis efficiency.

## Introduction

Periodic fever, aphthous stomatitis, pharyngitis, and cervical adenitis syndrome (PFAPA) is an autoinflammatory disease that is characterized by clockwork-like, regularly occurring episodes of high fever, lasting 3–7 days and recurring every 3–5 weeks (1). During a fever episode, patients experience one or more of the symptoms, including pharyngitis, oral aphthous lesions, and cervical adenitis, accompanied by elevated inflammatory markers. Diagnosis is often delayed because of the lack of specific biomarkers and some patients did not have typical features (2), leading to difficulty in distinguishing symptoms between PFAPA and acute bacterial infection, such as pneumonia, tonsillitis or pyelonephritis, causing the overuse of antibiotics.

Although the fever episodes of PFAPA are self-limited, many patients may be treated with repeated courses of antibiotics, resulting in direct costs for medications and indirect costs related to medical consultations (3). Thus, the accurate and timely diagnosis of PFAPA is important; rapid and non-invasive biomarkers will be required in the early diagnosis of PFAPA. It was reported that the level of IFN-γ during a fever attack in patients with PFAPA increased markedly compared to patients with recurrent tonsillitis (4). However, the spectrum of serum cytokines and other laboratory parameters in the differential diagnosis between PFAPA and bacterial infection remain unknown. The exploration of the differences is crucial to promote early diagnosis and further optimize clinical management.

We performed a single-center, retrospective study to describe the clinical manifestations of patients with PFAPA and to assess the potential role of cytokine profiles and other laboratory parameters during an episode of high fever in the early diagnosis of this disease.

## Methods

### Patient cohort and study approval

Patients with PFAPA or identified bacterial infection were retrospectively enrolled from January 2020 to June 2024 in Shenzhen Children’s Hospital affiliated Shantou University Medical College. This study was approved by the ethics committees at the Shenzhen Children’s Hospital in China. Ethical approval was obtained, and a waiver of re-obtaining written informed consent was granted as the research involved minimal risk and used anonymized retrospective data.

We retrospectively included two groups of patients (PFAPA and bacterial infection) who had cytokine panels performed by Flowcytomix technique at admission during the febrile episodes, prior to any immunomodulatory medication therapy, with these results available in the electronic medical record. None of the patients were enrolled in any prospective study. The diagnosis of PFAPA syndrome was made according to the Eurofever/PRINTO classification criteria (5) and all these patients with PFAPA also met the definition proposed by CARRA PFAPA work group (6). To exclude common monogenic autoinflammatory diseases, most PFAPA patients underwent targeted genetic testing or whole-exome sequencing, including analysis of MEFV, MVK, TNFRSF1A, NLRP3, and other relevant genes. None of the participants were found to carry pathogenic variants associated with known monogenic autoinflammatory syndromes. Patients with identified bacterial infection were defined by a clinically evident focus corroborated by positive cultures (blood, urine, or other sterile sites), and/or supportive laboratory findings, including markedly elevated CRP, procalcitonin, or white blood cell count.

### Data collection

Clinical data were collected from inpatient medical records, including clinical manifestations, laboratory findings, and levels of serum cytokines. Laboratory findings included white blood cell (WBC) counts, neutrophil counts, platelet counts, erythrocyte sedimentation rate (ESR), levels of hemoglobin, serum amyloid A (SAA), C-reactive protein (CRP), ferritin, and fibrinogen. Serum cytokines included IFN-γ, IL-10, IL-6, TNF-α, IL-4, and IL-2.

### Statistical analysis

Statistical analysis was performed in SPSS version 24.0 (SPSS, Inc.,Chicago, IL, USA) and GraphPad Prism software (version 8.0.1, GraphPad Inc). Continuous variables were expressed as medians with interquartile ranges and categorical variables were presented as percentages. Unpaired Welch’s t-test and Mann-Whitney U test for continuous data were performed. Categorical variables were compared using the chi-square test or Fisher’s exact test. Sensitivity, specificity, positive and negative predictive values, as well as the connection between the cutoff value and the actual classification, were examined and expressed via receiver operating curve (ROC) analysis. A value of p<0.05 was considered statistically significant. Variables found to be statistically significant on univariate analysis were included in binary logistic regression analysis. Stepwise logistic regression with classical model selection was employed to identify PFAPA. Model calibration was assessed using the Hosmer–Lemeshow goodness-of-fit test, where a p-value > 0.05 indicated adequate fit. Nagelkerke R^2^ was used to estimate the model’s explanatory power. Classification accuracy was also reported.

## Results

### Clinical and laboratory characteristics in study groups

A total of 227 children were enrolled in the study, including 67 patients with PFAPA and 160 patients with identified bacterial infection. Clinical characteristics and laboratory findings were summarized and compared between the two groups in **Table 1**. There were no significant differences in the distribution of gender and age between the two groups (all *P* > 0.05). Patients with PFAPA experienced a significantly longer time to diagnosis from the onset of symptoms compared to those with bacterial infection, with the median delay in the PFAPA group being 12 months (*P* < 0.0001). The median time from fever onset to sampling was shorter in PFAPA patients compared to those with identified bacterial infection (*P* < 0.0001). A higher proportion of patients with bacterial infection experienced cervical lymphadenopathy (*P* = 0.0367) and skin rashes (*P* < 0.0001) compared to those with PFAPA. However, no significant differences were observed in other clinical manifestations between the two groups, including arthralgia, respiratory symptoms, hepatosplenomegaly, etc (all *P* > 0.05)(**Table 1**).

**Table 1:** Comparison of clinical and laboratory parameters in patients with PFAPA syndrome and identified bacterial infection.

Characteristics, median (IQR)	PFAPA (n=67)	Identified bacterial infection (n=160)	*P* value
Gender (Female/Male)	34/33	60/100	0.0646
Age at symptom onset (years)	4.7 (2.7, 7.3)	5.3 (2.5, 8.2)	0.8075
Sampling day after the onset of fever (days)	2 (2, 3)	6 (4, 8)	***<0.0001***
Disease onset to diagnosis (months)	12 (8, 24)	0.2 (0.1, 0.3)	***<0.0001***
Rash (%)	3 (4.5)	48 (30)	***<0.0001***
Cervical lymphadenopathy (%)	42 (62.7)	76 (47.5)	***0.0367***
Respiratory menifestations (%)	26 (38.8)	82 (51.3)	0.1089
Gastrointestinal manifestations (%)	14 (20.9)	50 (31.3)	0.1454
Arthralgia (%)	1 (1.5)	11 (6.9)	0.0983
Hepatomegaly (%)	6 (9.0)	30 (18.8)	0.065
Splenomegaly (%)	3 (4.5)	10 (6.3)	0.600
White blood cell (×109/L)	10.7 (8.9, 13.3)	11.6 (7.9, 16.9)	0.4768
Neutrophils (×109/L)	7.5 (6.1, 9.6)	7.2 (4.8, 12.3)	0.835
Hemoglobin (g/L)	115 (109, 119)	114 (104.3, 121)	0.5659
Platetet (×109/L)	248 (212, 278)	300 (223.5, 378)	***0.0002***
C-reactive protein (mg/L)	41.1 (21.4, 71.2)	49.4 (21.8, 84.8)	0.4738
Erythrocyte sedimentation rate (mm/h)	28 (18, 36)	40 (25, 62)	***<0.0001***
Serum amyloid A (mg/L)	113.2 (53.15,350.2)	116.5 (54.71,414.5)	0.547
Ferritin (ng/mL)	101 (77, 148)	189 (117, 312)	***<0.0001***
Fibrinogen (g/L)	4.5 (3.7, 4.9)	5 (4, 6.4)	***0.0005***
Interleukin-2 (pg/mL)	0.7 (0, 2.2)	1.1 (0.2, 2.0)	0.3543
Interleukin-4 (pg/mL)	1.5 (1.0, 2.5)	1.7 (1.1, 2.6)	0.6358
Interleukin-6 (pg/mL)	25.1 (2.8, 65)	27.6 (13.2, 66.8)	0.7223
Interleukin-10 (pg/mL)	2.6 (1.3, 4.3)	3.5 (2.3, 7.2)	***0.0016***
Tumor necrosis factor-α (pg/mL)	2.4 (0.9, 3.1)	1.9 (0.7, 3.2)	0.323
Interferon-γ (pg/mL)	16.3 (5.6, 40.8)	3.2 (1.7, 7.7)	***<0.0001***
IFN-γ/IL-6	0.7 (0.28, 1.2)	0.2 (0, 0.5) ***<0.0001***	

PFAPA, periodic fever, aphthous stomatitis, pharyngitis, and cervical adenitis; IQR, interquartile range; IFN-γ, interferon-γ; IL-6, interleukin-6.

Bold represents statistically significant indicators.

**Figure 1:**
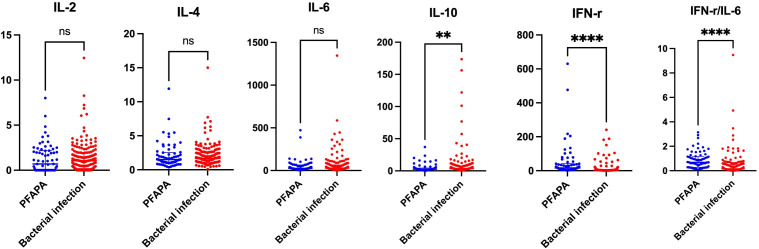
Serum levels of cytokines in patients wit PFAPA and bacterial infection.

No significant differences were observed in WBC counts, neutrophil counts, or levels of hemoglobin, SAA and CRP between the two groups (all *P* > 0.05). In comparison to patients with identified bacterial infection, patients with PFAPA had lower platelets counts (*P* = 0.0002) and reduced levels of ESR (*P* < 0.0001), ferritin (*P* < 0.0001), fibrinogen (*P* = 0.0005), and serum IL-10 (*P* = 0.0016). The median level of serum IFN-γ in patients with PFAPA was 16.3 pg/mL, and the median IFN-γ/IL-6 ratio was 0.7, both of which were significantly higher than those observed in patients with bacterial infection (*P* < 0.0001). No significant differences were found in the levels of other serum cytokines, including TNF-α, IL-2, IL-6, and IL-4 (all *P* > 0.05)(**Figure 1**, **Table 1**).

### The potential role of serum cytokines in the diagnosis of PFAPA

The multivariable logistic regression model included variables with a *P*-value <0.05 in univariate analysis and minimal collinearity. The analysis revealed that PLT, IL-10, and the IFN-γ/IL-6 ratio were independent predictors of patients with PFAPA (**Table 2**). The model demonstrated excellent discriminative ability (Nagelkerke R^2^ = 0.736), a statistically significant overall fit (*P* < 0.001), and acceptable calibration (Hosmer-Lemeshow test *P* = 0.842). According to this model, the correct classification rate for the PFAPA group was 83.9%, with an overall accuracy rate of 88.8%.

**Table 2:** Multiple logistic regression analysis of parameters in PFAPA syndrome and identified bacterial infection.

Indicators	B	SE	*P* value	OR	95% CI
Sampling day after the onset of fever (days)	-0.831	0.174	***<0.001***	0.435	0.31-0.613
Rash (%)	-1.373	0.79	0.082	0.253	0.054-1.192
Cervical lymphadenopathy (%)	1.622	0.582	0.005	5.062	1.617-15.851
Platetet (×109/L)	-0.011	0.004	***0.002***	0.989	0.982-0.996
Erythrocyte sedimentation rate (mm/h)	-0.037	0.02	0.063	0.964	0.927-1.002
Ferritin (ng/mL)	-0.005	0.003	0.086	0.995	0.990-1.001
Fibrinogen (g/L)	0.157	0.271	0.563	1.17	0.687-1.992
Interleukin-10 (pg/mL)	-0.082	0.027	***0.003***	0.921	0.873-0.972
Interferon-γ (pg/mL)	-0.001	0.004	0.829	0.999	0.991-1.007
IFN-γ/IL-6	1.075	0.43	***0.012***	2.93	1.261-6.808

Accurancy rate: PFAPA: 83.9%, Bacterial infection: 91.1%, General: 88.8%.

B, regression coefficients; CI, confidence interval; SE, standard error.

Bold represents statistically significant indicators.

**Figure 2:**
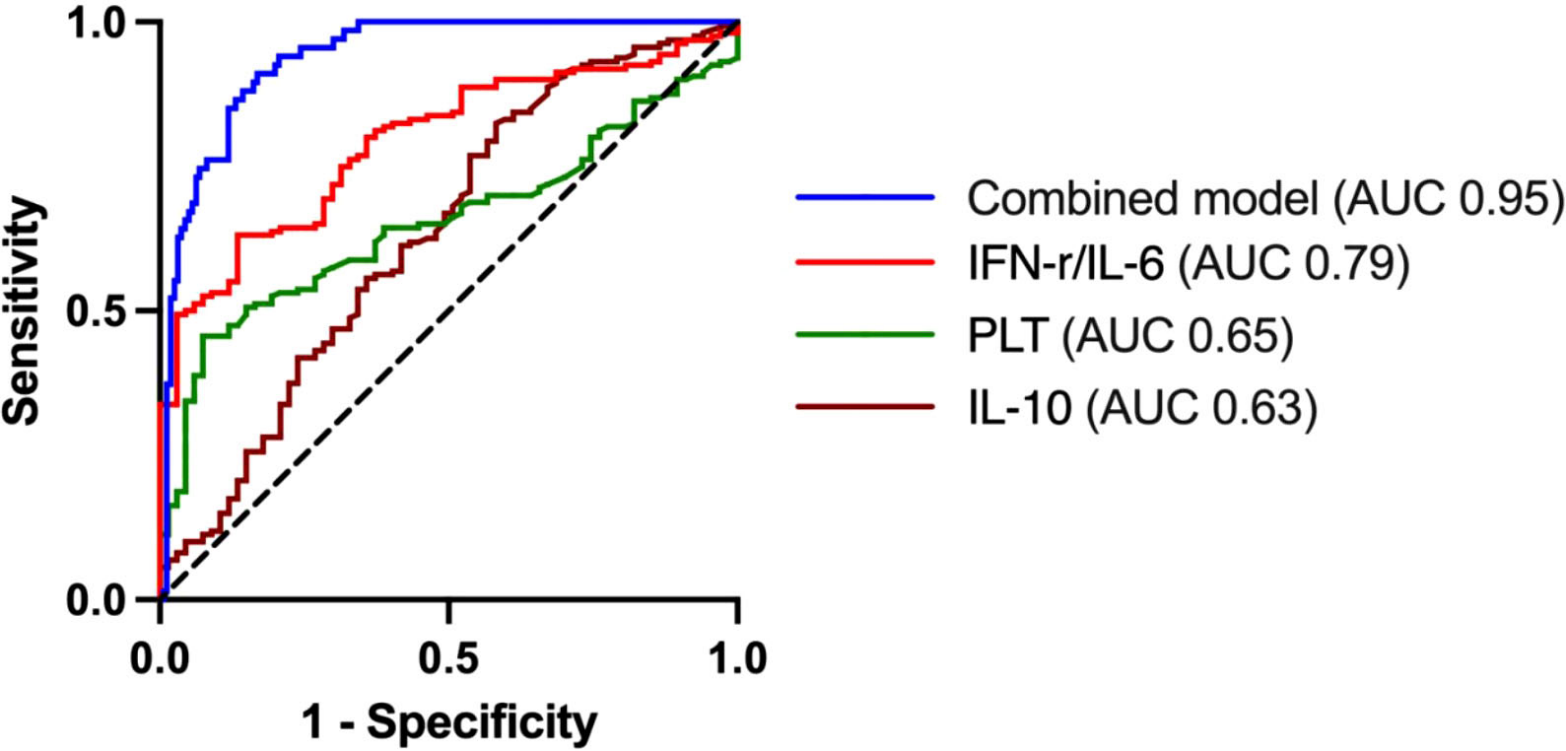
Receiver operating characteristic curves of combined model and the role of IFN-γ/IL-6, PLT and IL-10.

A ROC curve analysis to assess the potential role of serum cytokines and laboratory parameters in the diagnosis of PFAPA was given in **Table 3**. The cutoff value for the serum IFN-γ/IL-6 ratio to differentiate PFAPA from bacterial infection was 0.43 (AUC, 0.79, 95%CI: 0.73–0.85). With a threshold of IFN-γ/IL-6 > 0.43, the sensitivity and specificity were 70.15% and 71.88%, respectively. Moreover, the combined diagnostic model demonstrated an AUC of 0.95 (95% CI: 0.93–0.98), with a cut-off value of 0.38. The model demonstrated higher sensitivity (90.3%) and specificity (89.6%), significantly improving diagnostic accuracy (**Figure 2**).

**Table 3:** Receiver operating characteristic curve analysis of laboratory parameters in two groups.

Indicators	Cutoff	Sensitivity (%)	Specificity (%)	95% CI	AUC	*P* value
Platetet (×109/L)	258	61.19	64.38	0.58-0.72	0.65	0.0003
Interleukin-10 (pg/mL)	3.2	62.69	55.63	0.55-0.71	0.63	0.0017
IFN-γ/IL-6	0.43	70.15	71.88	0.73-0.85	**0.79**	<0.0001
Combined model	0.38	90.3	89.6	0.93-0.98	**0.95**	<0.0001

AUC, area under the ROC curve.

Bold represents statistically significant indicators.

## Discussion

The diagnosis of PFAPA is primarily based on clinical characteristics, and there is no definitive diagnostic test available. Significant delays in diagnosis have been observed in both developed and developing countries (7, 8). In our cohort, the median interval between onset and diagnosis was 12 (8, 24) months. PFAPA primarily affects young children, manifesting with recurrent fever and pharyngitis. It is frequently misdiagnosed as recurrent streptococcal pharyngitis, leading to unnecessary antibiotic treatments (9). Overuse of antibiotics results not only in direct costs for medications but also contributes to the growing problem of bacterial resistance (10). Additionally, excessive antibiotic use carries the risk of drug-related side effects, disrupts the gut microbiota, and may result in long-term health consequences (11). Rydenman et al (3). had demonstrated that the annual number of antibiotic prescriptions for PFAPA patients decreased from 2.1 before diagnosis to 0.8 after diagnosis. Therefore, early diagnosis of PFAPA is crucial for reducing unnecessary antibiotic use.

Patients with PFAPA were less likely to experience rash and cervical lymphadenopathy compared to those with bacterial infections. However, no significant differences were observed in other clinical symptoms or common laboratory parameters such as CRP, WBC, and neutrophils. This study underscored the difficulty in distinguishing PFAPA and bacterial infection according to clinical symptoms. In our cohort, the median time from fever onset to cytokine sampling was shorter in patients with PFAPA compared to those with identified bacterial infection. The earlier sampling in PFAPA likely reflected a higher clinical suspicion of autoinflammatory disease, as physicians tended to request cytokine test sooner in children with recurrent fever of unclear etiology. Consequently, this heightened clinical suspicion likely influenced the timing of the blood draws. To reduce potential bias arising from differences in sampling times, we conducted an additional analysis restricted to samples collected within a 24–72 hour window, ensuring comparable sampling times between the two groups. The results remained consistent, showing that both IFN-γ levels and the IFN-γ/IL-6 ratio reliably distinguished the two groups (**Supplementary Table 1**). These findings indicate that the observed differences in cytokine profiles are robust and not attributable to variability in sample collection timing.

It was reported that IFN-induced genes (AIM2, CXCL10) were significantly overexpressed during PFAPA attacks, with flares associated with high levels of proinflammatory cytokines (IL-18 and IL-6) (12). Kelly et al. (13) also demonstrated that IFN-γ induced cytokine (IP-10/CXCL10) increased after fever onset in PFAPA patients. In our study, PFAPA patients exhibited an obvious increase in levels of IFN-γ and the IFN-γ/IL-6 ratio, but no significant difference in the level of IL-6 was observed. In contrast, septic patients with bacterial co-infection have been reported to have elevated levels of IL-6 and TNF-α (14). Similarly, Qiuhua Zhu et al. (15) also reported that elevated IL-6 and IL-10 are closely associated with the severity of bacterial bloodstream infections. Our findings align with this, showing that patients with bacterial infection had higher IL-10 level. Therefore, our findings suggested that an elevated IFN-γ/IL-6 ratio, rather than the level of IL-6 or IL-10, could be a more reliable marker to distinguish PFAPA from bacterial infection. ROC analysis revealed that the IFN-γ/IL-6 ratio, with a cut-off value of 0.43, yielded an AUC of 0.79 to predict PFAPA. We found that IL-10, PLT, and IFN-γ/IL-6 ratio were promising indicators to discriminate PFAPA and bacterial infection. Subsequently, we developed a model in combination of these biomarkers, which demonstrated an excellent AUC of 0.95. Thus, this diagnosis model may provide a robust and effective method to distinguish PFAPA from bacterial infection.

IFN-γ, predominantly produced by NK and T cells, is a pleiotropic cytokine with multiple effects on the inflammatory response and on innate and adaptive immunity (16). Based on this study, we speculated that the elevated level of IFN-γ during disease flare in PFAPA patients is associated with the activation T lymphocytes, especially Th1 cells, targeting inflamed peripheral tissues such as lymph nodes and adenoids (17). This suggested a dysregulated immune response in PFAPA, characterized by continuous activation of proinflammatory cytokine (12). IL-10, an anti-inflammatory cytokine, serves as a key negative regulator of the immune response, inhibiting the activity of proinflammatory cytokines. In patients with bacterial infection, elevated IL-10 level during febrile episodes indicated that the inflammatory response activated by pathogen-associated molecular patterns (PAMPs) or damage-associated molecular patterns (DAMPs) (18) was suppressed.

This single-center retrospective study was limited by its small sample size, the lack of samples collected during asymptomatic phases, and the potential effect of varying sampling times. To address these limitations, we are planning a large, multicenter prospective cohort study involving these two patient groups, in which samples will be collected at comparable time points during both flare and non-flare phases. This forthcoming study is designed to definitively validate the diagnostic utility of serum cytokine profiles in PFAPA syndrome.

## Conclusions

Our study identified IFN-γ/IL-6 ratio during febrile episodes as a potential marker in the early diagnosis of PFAPA, differentiating it from bacterial infection. The combined model incorporating IFN-γ/IL-6, IL-10, and PLT optimized the diagnosis efficiency. Early recognition of PFAPA patients could be beneficial to reduce unnecessary antibiotic use.

## Data Availability

The original contributions presented in the study are included in the article/**Supplementary Material**. Further inquiries can be directed to the corresponding author.
