# Supplementary material for: Cytokine profiles and laboratory parameters as indicators to distinguish children with PFAPA and bacterial infection
Source: Front Immunol. 2026 Feb 12;17:1683221. doi: 10.3389/fimmu.2026.1683221 (PMC12935951; doi:10.3389/fimmu.2026.1683221)
Supplement: Supplementary file 1 [file Table1.pdf]

Comparison of clinical and laboratory parameters in patients with PFAPA syndrome and identified bacterial infection

Characteristics, median (IQR), not specified	PFAPA (n = 55)	Identified bacterial infection (n = 34)	*P* value
Gender (Female/Male)	26/29	12/22	0.1089
Age at symptom onset (years)	4.1 (2.5, 6.3)	4.5 (2.5, 6.3)	0.6038
Sampling day after the onset of fever (days)	2 (2, 3)	2 (2, 3)	0.6887
Disease onset to diagnosis (months)	12 (8, 24)	0.1 (0.07, 0.13)	***<0.0001***
Rash (%)	2 (3.6)	10 (29.4)	***0.0005***
Cervical lymphadenopathy (%)	23 (41.8)	12 (35.3)	0.5404
Arthralgia (%)	1 (1.8)	1 (2.9)	0.7284
Hepatomegaly (%)	5 (9.1)	5 (14.7)	0.4151
Splenomegaly (%)	2 (3.6)	2 (5.9)	0.6192
White blood cell ($\times 10^9$/L)	10.9 (8.5, 13.6)	15.8 (10.3, 22.6)	***<0.0001***
Neutrophils ($\times 10^9$/L)	7.8 (6.3, 9.7)	12.6 (8.5, 17.1)	***0.0002***
Hemoglobin (g/L)	116 (111, 119)	115.5 (107.8, 121.5)	0.3249
Platetet ($\times 10^9$/L)	248 (207, 275)	301.5 (225, 405.5)	***0.0044***
C-reactive protein (mg/L)	38.3 (20.5, 71)	65.9 (33.0, 90.5)	0.0519
Erythrocyte sedimentation rate (mm/h)	27 (18, 33)	38 (23.5, 60)	***0.0013***
Serum amyloid A (mg/L)	103.7 (49.7,319.4)	222.9 (82.4,879.4)	0.0764
Ferritin (ng/mL)	96.4 (76, 147)	166.4 (115, 245.5)	***0.0004***
Fibrinogen (g/L)	4.3 (3.6, 4.7)	4.8 (3.8, 6.1)	***0.0193***
Interleukin-2 (pg/mL)	0.7 (0, 2.2)	1.3 (0, 2.0)	0.9983
Interleukin-4 (pg/mL)	1.4 (1.0, 2.2)	1.5 (1.0, 2.6)	0.9314
Interleukin-6 (pg/mL)	25.8 (14.7, 65)	50.6 (20.5, 99.8)	0.0532
Interleukin-10 (pg/mL)	2.7 (1.3, 5.2)	4.0 (2.3, 10.3)	***0.0243***
Tumor necrosis factor-α (pg/mL)	2.4 (0.9, 3.1)	1.5 (0.6, 2.9)	0.2245
Interferon-γ (pg/mL)	16.3 (5.9, 40.9)	2.9 (1.4, 11.7)	***<0.0001***
IFN-γ/IL-6	0.7 (0.3, 1.3)	0.1 (0, 0.4)	***<0.0001***

PFAPA: periodic fever, aphthous stomatitis, pharyngitis, and cervical adenitis; IQR: interquartile range; IFN-γ: interferon-γ; IL-6: interleukin-6
